# 2486: Sleep, biological stress, and health in a community sample of toddlers living in socioeconomically disadvantaged homes

**DOI:** 10.1017/cts.2017.235

**Published:** 2018-05-10

**Authors:** Monica Roosa Ordway, Nancy Redeker, Lois Sadler

**Affiliations:** Yale School of Medicine, New Haven, CT, USA

## Abstract

OBJECTIVES/SPECIFIC AIMS: The purposes of this study are to examine the relationships among sleep characteristics (duration, efficiency), stress biomarkers, and child behavior problems among toddlers living in socioeconomically disadvantaged homes and how these characteristics change over time from age of 12 months to 24 months. Aim 1: examine changes in subjective and objective sleep characteristics from 12 to 24 months of age. Aim 2: examine changes in stress biomarkers from 12 to 24 months of age. Aim 3: examine the cross sectional and longitudinal relationships between sleep characteristics and stress response. Aim 4: examine the cross sectional and longitudinal relationships between sleep characteristics and toddlers’ child behavior problems. METHODS/STUDY POPULATION: In this cross-sectional study we are recruiting parents with healthy toddlers from early head start programs and a community clinic to prospectively examine the relationships among sleep characteristics, stress biomarkers, and children’s health. Data on sleep characteristics will include subjective and objective measures of sleep duration and efficiency and parental interactive bedtime behaviors to assist their toddlers’ sleep initiation. Multisystemic biomarkers of stress including cortisol, CRP, IL-6, and BMI, will be measured individually. The associations between sleep characteristics and the biomarkers, considered as a latent variable of the stress response, will be explored. Health measures will include secretory IgA and parent-reported behavioral problems. Generalized linear models will be used in the data analysis. RESULTS/ANTICIPATED RESULTS: To date we have obtained objective (9 days/nights of actigraphy) measures of 33 toddlers’ sleep and subjective measures of parenting interactive behaviors. Using the Parental Interactive Bedtime Behavior (PIBB) Survey and subscales [active physical comforting, encourage autonomy, settle by movement, passive physical comforting (PPC), social comforting], we are currently reporting on the associations between PIBB and toddler’s sleep characteristics. The sample included 33 toddlers (mean age=1.33 years, SD=0.54). The toddlers’ sleep duration averaged 8.22 hours (SD=0.86). There were statistically significant moderate associations between sleep duration and parents’ PPC (*r*=−0.41, *p*=0.02). Intra-individual variability in the amount of wake after sleep onset was also significantly associated with total PIBB and PPC (*r*=0.37, *p*=0.05; *r*=0.52, *p*=0.002, respectively). Intra-individual variability in the amount of sleep fragmentation within toddlers was significantly associated with total PIBB (*r*=0.36, *p*=0.05). DISCUSSION/SIGNIFICANCE OF IMPACT: Although active physical comforting (eg, rocking to sleep, patting or rubbing child’s back) is most commonly associated with sleep patterns in infancy and toddlerhood among samples of higher socio-economic status, findings from this study suggest a stronger association between PPC (eg, presence of the parent in the room to fall asleep) and less sleep duration and more individual variability in night wakings. The biomarker data are currently being analyzed and results will be presented within the year. Taken together, these preliminary results and pending results will inform future intervention development that may address the role of parenting behavior in promoting health sleep early in life.

